# Acupuncture for Adults with Diarrhea-Predominant Irritable Bowel Syndrome or Functional Diarrhea: A Systematic Review and Meta-Analysis

**DOI:** 10.1155/2020/8892184

**Published:** 2020-11-22

**Authors:** Jianbo Guo, Xiaoxiao Xing, Jiani Wu, Hui Zhang, Yongen Yun, Zongshi Qin, Qingyong He

**Affiliations:** ^1^Guang'anmen Hospital, China Academy of Chinese Medical Sciences, Beijing, China; ^2^Beijing University of Chinese Medicine, Beijing, China; ^3^Henan University of Chinese Medicine, Henan, China; ^4^Faculty of Medicine, The University of Hong Kong, Hong Kong, China

## Abstract

*Obj*ective. To evaluate the clinical effectiveness and safety of acupuncture therapy in the treatment of diarrhea-predominant irritable bowel syndrome (IBS-D) or functional diarrhea (FD) in adults. *Method.* Five electronic databases—PubMed, EMBASE, CNKI, VIP, and Wanfang—were searched, respectively, until June 8, 2020. The literature of clinical randomized controlled trials of acupuncture for the treatment of IBS-D or FD in adults were collected. Meta-analysis was conducted by Using Stata 16.0 software, the quality of the included studies was assessed by the RevMan ROB summary and graph, and the results were graded by GRADE. *Result*. Thirty-one studies with 3234 patients were included. Most of the studies were evaluated as low risk of bias related to selection bias, attrition bias, and reporting bias. Nevertheless, seven studies showed the high risk of bias due to incomplete outcome data. GRADE's assessments were either moderate certainty or low certainty. Compared with loperamide, acupuncture showed more effectiveness in weekly defecation (SMD = −0.29, 95% CI [-0.49, -0.08]), but no significant improvement in the result of the Bristol stool form (SMD = −0.28, 95% CI [-0.68, 0.12]). In terms of the drop-off rate, although the acupuncture group was higher than the bacillus licheniformis plus beanxit group (RR = 2.57, 95% CI [0.24, 27.65]), loperamide group (RR = 1.11, 95% CI [0.57, 2.15]), and trimebutine maleate group (RR = 1.19, 95% CI [0.31, 4.53]), respectively, it was lower than the dicetel group (RR = 0.83, 95% CI [0.56, 1.23]) and affected the overall trend (RR = 0.93, 95% CI [0.67, 1.29]). Besides, acupuncture produced more significant effect than dicetel related to the total symptom score (SMD = −1.17, 95% CI [-1.42, -0.93]), IBS quality of life (SMD = 2.37, 95% CI [1.94, 2.80]), recurrence rate (RR = 0.43, 95% CI [0.28, 0.66]), and IBS Symptom Severity Scale (SMD = −0.75, 95% CI [-1.04, -0.47]). Compared to dicetel (RR = 1.25, 95% CI [1.18, 1.32]) and trimebutine maleate (RR = 1.35, 95% CI [1.13, 1.61]), acupuncture also showed more effective at total efficiency. The more adverse effect occurred in the acupuncture group when comparing with the dicetel group (RR = 11.86, 95% CI [1.58, 89.07]) and loperamide group (RR = 4.42, 95% CI [0.57, 33.97]), but most of the adverse reactions were mild hypodermic hemorrhage. *Conclusion.* Acupuncture treatment can improve the clinical effectiveness of IBS-D or FD, with great safety, but the above conclusions need to be further verified through the higher quality of evidence.

## 1. Introduction

Diarrhea-predominant irritable bowel syndrome (IBS-D) or functional diarrhea (FD) is a disease with high incidence rates, which affects the lives of people in China, America, and even the world, often accompanied by mental illness [1–3]. The main clinical manifestations of IBS-D and FD are passing water samples three or more times daily, accompanied by abdominal pain and discomfort [4, 5]. It was considered to be a functional disease closely related to the physiological or mental status of patients, but a gradually in-depth study of pathophysiological mechanisms can explain these symptoms [6]. Calprotectin and fecal lactoferrin both are markers of an inflammatory response in IBS-D or FD. In particular, the psychological symptoms and visceral hypersensitivity of IBS-D or FD patients have been shown to be closely related to parasympathetic dysfunction, which may affect the severity of the disease [7, 8].

At present, anticholinergic drugs, antispasmodic drugs, antimotility, and antidiarrheal drugs are commonly used to treat IBS-D and FD, but adverse effects include dizziness, nausea, vomiting, and even respiratory inhibition. It is difficult to obtain the satisfactory effect of these drugs in IBS-D and FD patients. Probiotics are effective and safe in IBS patients, but studies on the detection of strains, dose, and duration of treatment are inconsistent. [9] Therefore, it is particularly important to find a treatment method that can effectively reduce pain in patients with fewer side effects [10].

Acupuncture, as a special nondrug technology in traditional Chinese medicine, is used to treat diseases by inserting fine needles or stimulating acupoints manually [11]. Previous studies have found that acupuncture treatment is closely related to the central nervous system and the intestinal nervous system; besides, acupuncture points cover the main nerve bundles of the body [12]. Evidence suggests that acupuncture can produce curative effects on gastrointestinal motility through nerve and body fluid channels [13–17]. This study explores the effectiveness and safety of acupuncture in the treatment of IBS-D or FD by systematic review and meta-analysis.

## 2. Methods

### 2.1. Search Strategy

This meta-analysis was conducted by guidelines [18, 19] set out in the PRISMA statement (Supplementary material 1: PRISMA Checklist) and was registered with PROSPERO (CRD42015017574). We conducted a literature search (using PubMed), the Chinese Science and Technology Periodical Database (Embase), the Chinese National Knowledge Infrastructure Database (CNKI), China Scientific Journal Database (VIP), and Wanfang Database. The retrieval time was from the establishment of the database to June 8, 2020. The search method combined MeSH subject words and free search words as follows: “diarrhea OR irritable bowel syndrome OR functional diarrhea” AND “acupuncture” AND “randomly” AND “controlled.” Supplementary material 2 outlines the search strategy of the PubMed database. This study protocol has been published previously [Qin et al. 2018].

### 2.2. Inclusion and Exclusion Criteria

The literature included in our study met the following requirements: (1) study type: clinical randomized controlled trials of acupuncture treatment for IBS-D or FD, blinded or nonblinded, written in Chinese or English, and available online before June 8, 2020; (2) intervention measures: the treatment group was treated with penetrating acupuncture, or combined with a control group, and the control group was treated with conventional medicine, sham acupuncture, or conventional acupuncture; (3) participants: patients aged 18 years and over, with unlimited gender and case source, who were definitively diagnosed with IBS-D or FD; and (4) outcome indicators: weekly defecation rate, patient drop off rate, Bristol stool form, total symptom score, IBS quality of life (IBS-QOL), total efficiency, recurrence rate, IBS Symptom Severity Scale (IBS-SSS) and adverse effect. The exclusion criteria were as follows: (1) studies of non-IBS-D or FD cases; (2) the intervention measures of the treatment group were nonpenetrating acupuncture, such as laser acupuncture, acupoint pressing, percutaneous, or percutaneous electrical nerve stimulation; (3) the control group and the experimental group were used for different types of acupuncture (i.e., acupuncture and electroacupuncture); (4) conference papers; (5) the literature on the effectiveness evaluation index did not meet the inclusion requirements; (6) literature published multiple times; and (7) literature with Western medicine or other therapies as the main research objective.

### 2.3. Literature Quality Assessment

According to the Cochrane criteria, we assessed the quality of the included studies in six domains: (1) random treatment assignment; (2) treatment assignment concealment; (3) treatment blinding (including blinding for patients, study implementers, and study outcome assessors); (4) data integrity of the study results; (5) selective reporting in the study; and (6) other biases. From the above domains, two researchers (J.G and X.X) evaluated the risk of bias in the included literature according to the three criteria of “low risk,” “high risk,” or “unknown risk.” In case of disagreement during the evaluation, the decision was made through consultation or discussion with a third researcher (Z.Q). GRADE (grades of recommendation, assessment, development, and evaluation) was used to grade and evaluate weekly defecation, Bristol stool form, total symptom score, IBS-QOL, and IBS-SSS analysis results.

### 2.4. Data Extraction and Analyses

Data extraction included (1) basic information of the study including the first author, year of publication, study time, sample size, and patient age; (2) treatment information of the study including treatment methods, outcome indicators, and adverse events, of the observation group, and the control group. If the data included in the study were incomplete, we tried to contact the original author for supplementation.

Stata 16.0 software was used for data analysis. A random-effect model was used, as different acupuncture points or intervention cycles in each study may affect the therapeutic effect. Cohen's *d* and 95% confidence interval (CI) were used for continuous variables, and RR (relative risk) was used for secondary variables. *Q* statistics and *I*^2^ were used to judge the heterogeneity of the study (i.e., when the *P* value of *Q*statistics < 0.1 or *I*^2^ > 50%, there is a large heterogeneity between the studies). A L'Abbe's chart was used to test the heterogeneity of binary variables. A meta-regression method and a bubble chart were used to evaluate the impact of related factors on outcome indicators and determine the source of heterogeneity. A funnel graph and an Egger test were used to evaluate publication bias. Finally, if there was significant heterogeneity between studies, a sensitivity analysis was conducted, and then meta-analysis was conducted by excluding the studies that induced heterogeneity.

## 3. Result

### 3.1. Literature Selection

**Figure 1:**
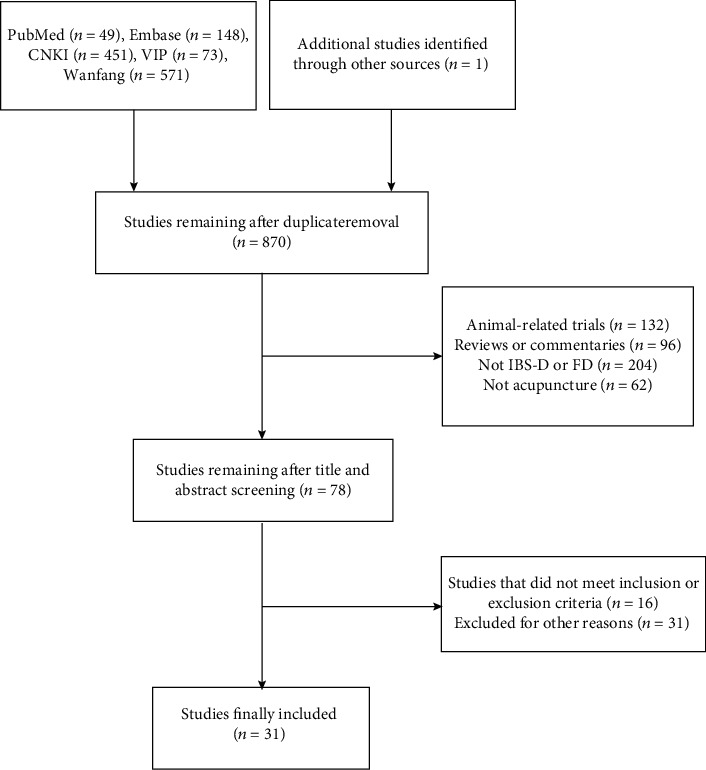
Flow chart of literature search.

Altogether, 1293 documents were retrieved, 870 of which were obtained after removing multiples of the same publication or publications with the same data, 78 of which were left after reading the title and abstract to address the inclusion criteria. After reading the full text, 31 studies met the inclusion standards and were finally included, all of which were published in journals.  Figure 1 shows the inclusion and exclusion flow chart.

### 3.2. Literature Characteristics

**Figure 2:**
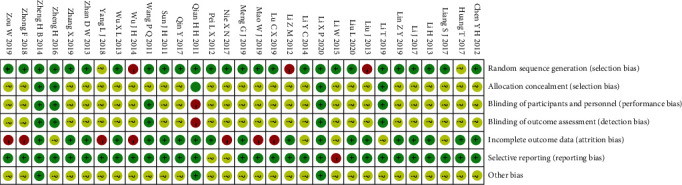
Risk of bias summary.

**Figure 3:**
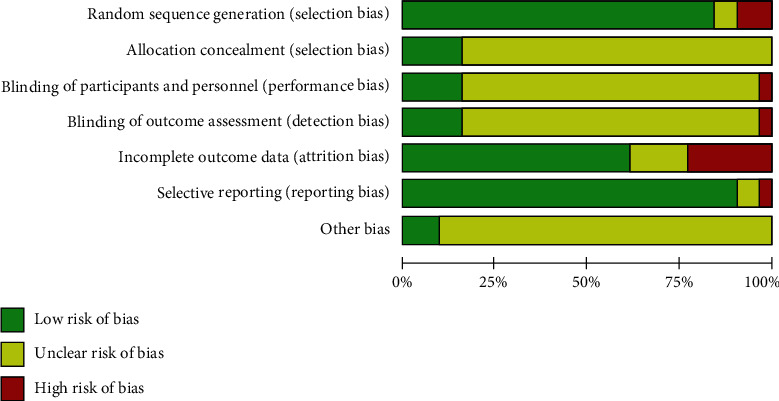
Risk of bias graph.

Among the 31 studies [20–50] included, 5 studies [24, 28, 30, 38, 41] used the random allocation method, which was evaluated as high risk or unknown risk; 5 studies [20, 32, 34, 47, 50] used the allocation hidden method, which was evaluated as low risk; 5 studies [22, 32, 34, 48, 50] used the blind method, which was evaluated as low risk; 12 studies [30, 31, 33, 34, 37, 39–42, 45, 46, 48] did not mention the completeness of the results, so were evaluated as high risk or unknown risk; 3 studies [25, 33, 37] did not use the selective report and were evaluated as a high risk or unknown risk; 3 studies [20, 32, 50] did not have any significant other sources of bias.  Table 1 presents the basic information about the included studies. Figures 2 and 3 present the risk of bias summary and graph related to the included studies, respectively. 26 studies reported methods of random sequence generation that were evaluated as low risk of bias, but 3 studies used nonstandard random grouping methods existed at the high risk of bias. As to allocation concealment of selection bias, performance bias, and detection bias, evaluations of numerous studies were regarded as unclear risk of bias. 18 studies with complete outcome data were evaluated as low risk of bias, but 7 studies existed at the high risk of bias due to incomplete outcome data. 28 studies with rarely selective reporting were evaluated as low risk of bias, and other biases in most of the included studies were unclear.  Table 2 presents the results of GRADE: weekly defecation, Bristol stool form, total symptom score, IBS-QOL, and IBS-SSS.

(1): weekly defecation; (2): patient drop-off rate; (3): Bristol stool form; (4): total efficiency; (5): IBS-QOL; (6): total symptom score; (7): recurrence rate; (8): IBS-SSS; (9): adverse reactions; NR: not reported; T: treatment group; C: control group

RCTs: randomized controlled trials; LOW (low certainty): our confidence in the effect estimate is limited: the true effect may be substantially different from the estimate of the effect; MODERATE (moderate certainty): we are moderately confident in the effect estimate: the true effect is likely to be close to the estimate of the effect, but there is a possibility that it is substantially different.

## 4. Result of Meta-Analysis

### 4.1. Weekly Defecation

**Figure 4:**
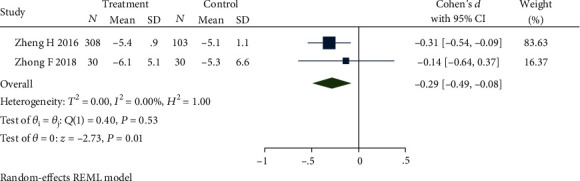
Forest plot of comparison of weekly defecation between the acupuncture group and loperamide group.

Four studies [32–34, 40] reported participants' number of defecations every week after treatment. The intervention methods of the control group were all loperamide. The results of the heterogeneity test demonstrated that there was no statistical significance (*P* = 0.19) between the studies and a significant heterogeneity existed between the studies (*Q*(3) = 24.65, *P* ≤ 0.01, *I*^2^ = 96.03%). Sensitivity analysis and meta-analysis removed studies [32, 33] that lead to this heterogeneity. One study [32] applied the form of electroacupuncture as the intervention which was different from comparative studies caused heterogeneity. The other study [33] selected fewer acupoints than comparative caused the heterogeneity. The updated forest plot is shown in Figure 4, demonstrating no heterogeneity among studies (*Q*(1) = 0.40, *P* = 0.53, *I*^2^ = 0.00%), while maintaining a statistically significant difference between studies (SMD = −0.29, 95% CI [-0.49, -0.08], *P* = 0.01).

### 4.2. Patient Drop-off Rate

**Figure 5:**
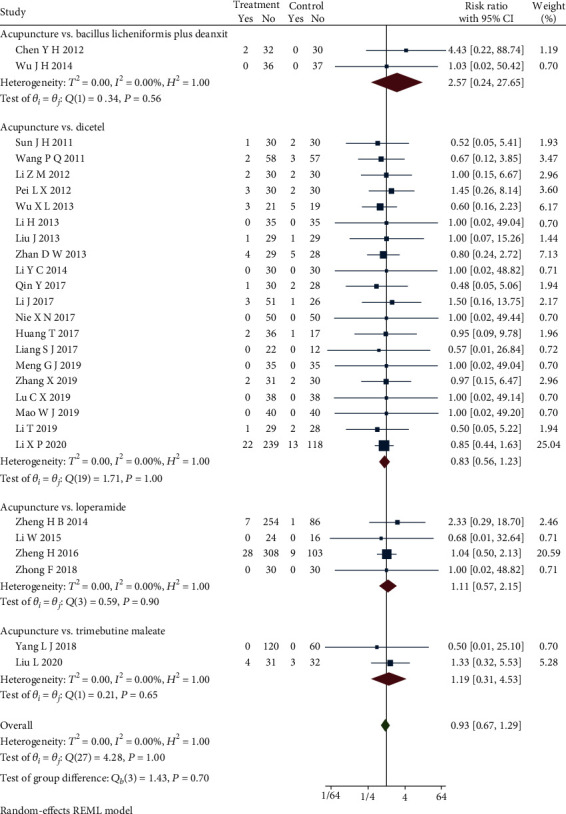
Forest plot of subgroup analysis on the patient drop-off rate.

**Figure 6:**
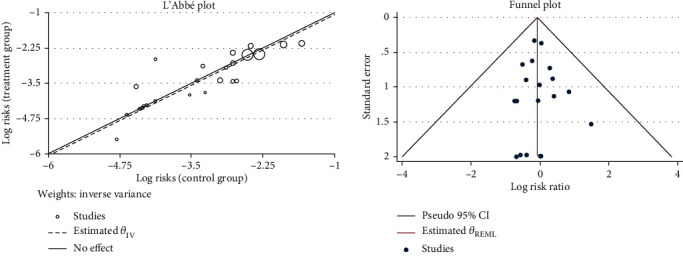
L'Abbe and funnel plots of the patient drop-off rate.

Thirty-one studies [20–50] reported the patient drop-off rate. Due to the different intervention methods of the control groups, we compared and analyzed some of the studies [21–41, 43–46, 48–50] through the subgroup. The results of the heterogeneity test showed that there was no significant difference in comparing the acupuncture group with the bacillus licheniformis plus deanxit group (RR = 2.57, 95% CI [0.24, 27.65], *P* > 0.05), the acupuncture group with the dicetel group (RR = 0.83, 95% CI [0.56, 1.23], *P* > 0.05), the acupuncture group with the loperamide group (RR = 1.11, 95% CI [0.57, 2.15], *P* > 0.05), the acupuncture group with the trimebutine maleate group (RR = 1.19, 95% CI [0.31, 4.53], *P* > 0.05), and no heterogeneity between these studies (*Q*(27) = 4.28, P = 1.00, *I*^2^ = 0.00%).  Figure 5 presents this data in a forest plot. Combined with shear complement analysis, Egger test results showed that there is no published bias (*β*_1_ = 0.03, SE of *β*_1_ = 0.35, *z* = 0.10, *P* = 0.92). The L'Abbe plot of the heterogeneity test and funnel plot are presented in Figure 6.

### 4.3. Bristol stool form

**Figure 7:**
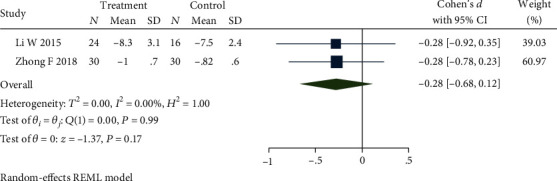
Forest plot of comparison of the Bristol stool form between the acupuncture group and loperamide group.

Four studies [32–34, 40] reported the stool form using Bristol's chart, and the intervention methods of the control group were all loperamide. The result showed that there was no statistical significance between studies (*P* = 0.31), but an obvious heterogeneity between the studies (*Q*(3) = 790.23, *P* ≤ 0.01, *I*^2^ = 99.91%). Sensitivity analysis and then a meta-analysis were carried out by removing studies [32, 34] that lead to this heterogeneity. One study [32] caused the heterogeneity still from the difference in acupuncture and electroacupuncture, and the other study [34] applied a different scoring method that resulted in heterogeneity.  Figure 7 presents a forest map demonstrating no heterogeneity among studies (*Q*(1) = 0.00, *P* = 0.17, *I*^2^ = 0.00%), and that there is no statistical significance between studies (SMD = −0.28, 95% CI [-0.68, 0.12], *P* = 0.17).

### 4.4. Total Symptom Score

**Figure 8:**
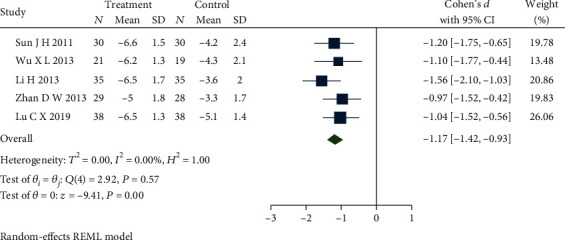
Forest plot of comparison of the total symptom score between the acupuncture group and dicetel group.

Seven studies [20, 21, 26, 27, 29, 42, 45] reported the total symptom score. The meta-analysis was completed by removing the studies [20, 42] which caused the high heterogeneity. One study [20] applied acupuncture plus dicetel as an intervention different from comparative studies, which could cause the heterogeneity. The other study [42] selected warm acupuncture as an intervention that could still cause heterogeneity.  Figure 8 presents a forest plot, which demonstrates no heterogeneity (*Q*(4) = 2.92, *P* = 0.57, *I*^2^ = 0.00%) among the studies which used dicetel in control groups, and that the differences among studies continue to be significantly different (SMD = −1.17, 95% CI [-1.42, -0.93], *P* ≤ 0.01). Across studies, the total score of symptoms in the treatment group was lower than that in the control group. Combined with the shear and complement analysis, Egger test results demonstrate that there was no publication bias (*β*_1_ = −0.16, SE of *β*_1_ = 4.37, *z* = −0.04, *P* = 0.97).

### 4.5. IBS-QOL

**Figure 9:**
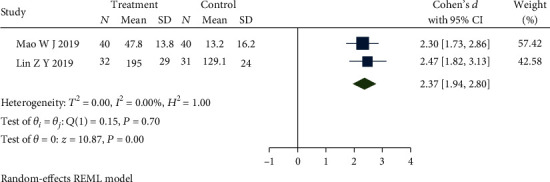
Forest plot of comparison of IBS-QOL between the acupuncture group and dicetel group.

Four studies [21, 29, 46, 47] reported the IBS-QOL and the intervention methods of the control group were all dicetel. The results showed that there was a significant statistical difference among studies (*P* ≤ 0.01) but obvious heterogeneity between studies (*Q*(3) = 32.75, *P* ≤ 0.01, *I*^2^ = 90.95%). After meta-analysis and eliminating studies [21, 29] which selected a different scoring method leading to this heterogeneity, the forest plot presented in Figure 9 demonstrates that there is no heterogeneity (*Q*(1) = 0.15, *P* = 0.7, *I*^2^ = 0.00%) among studies, and the differences between studies remain statistically significant (SMD = 2.37, 95% CI [1.94, 2.80], *P* ≤ 0.01). The quality of life in the treatment group was better than that in the control group.

### 4.6. Total Efficiency

**Figure 10:**
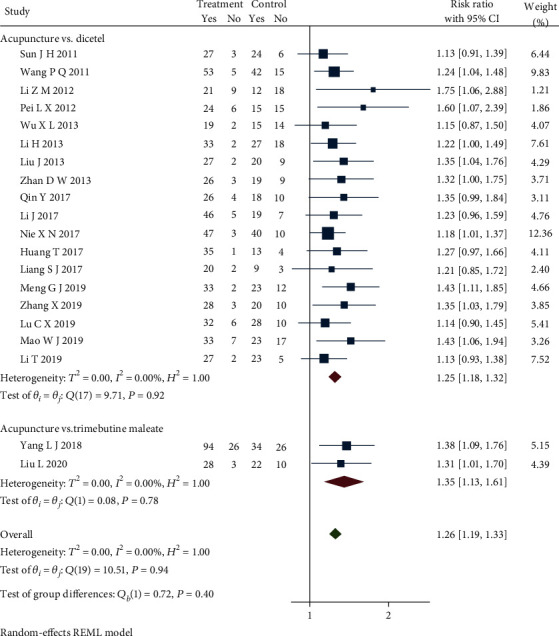
Forest plot of subgroup analysis on total efficiency.

**Figure 11:**
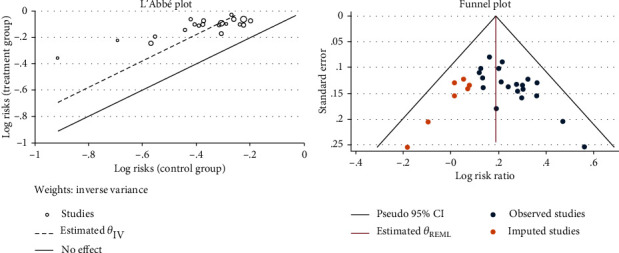
L'Abbe and funnel plots of total efficiency.

Twenty-seven studies [20–30, 26–30, 33, 35–39, 41–49] reported the total effective treatment rate. We can analyze 22 studies [21–30, 35–39, 41, 43–46, 48, 49] through the subgroup because of different western medicine in control groups. Sensitivity analysis removed two studies [23, 30] which caused obvious heterogeneity in a subgroup. One study applied electroacupuncture as an intervention, and the other study applied warm acupuncture that could cause heterogeneity. Updated subgroup analysis showed a significant statistical difference in comparing the acupuncture group with dicetel (RR = 1.25, 95% CI [1.18, 1.32], *P* < 0.05), the acupuncture group with the trimebutine maleate group (RR = 1.35, 95% CI [1.13, 1.61], *P* < 0.05), the acupuncture group with the pinaverium bromide tablet group (RR = 1.40, 95% CI [1.16, 1.69], *P* < 0.05), and no heterogeneity among studies (*Q*(19) = 10.51, *P* = 0.94, *I*^2^ = 0.00%).  Figure 10 presents the forest plot of the results. The total effective rate of the treatment group was greater than that of the control group. Combined with the shear complement analysis, funnel plots demonstrated that 7 published studies were missing. The Egger test showed that there were published biases (*β*_1_ = 1.98, SE of *β*_1_ = 0.90, *z* = 2.21, *P* = 0.03). The L'Abbe plot of the heterogeneity test and funnel plot both are shown in Figure 11.

### 4.7. Recurrence Rate

**Figure 12:**
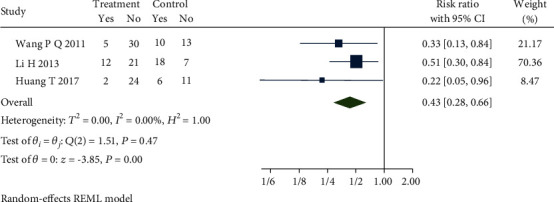
Forest plot of comparison of the recurrence rate between the acupuncture group and dicetel group.

Four studies [22, 23, 27, 38] reported the recurrence rate. Sensitivity analysis and then a meta-analysis were carried out by removing the study [23] which used a different oral medication that could cause the obvious heterogeneity in the control group.  Figure 12 presents the forest plot, which demonstrates that there is no heterogeneity (*Q*(2) = 1.51, *P* = 0.47, *I*^2^ = 0.00%) among the studies which used dicetel in control groups, and the differences between the studies remain statistically significant (RR = 0.43, 95% CI [0.28, 0.66], *P* ≤ 0.01). The recurrence rate of the treatment group was lower than that of the control group. Combined with the shear and complement analysis, there were two missing published biases in the funnel plot. Egger test results show that there is no published bias (*β*_1_ = −1.78, SE of *β*_1_ = 1.46, *z* = −1.22, *P* = 0.22).

### 4.8. IBS-SSS

**Figure 13:**
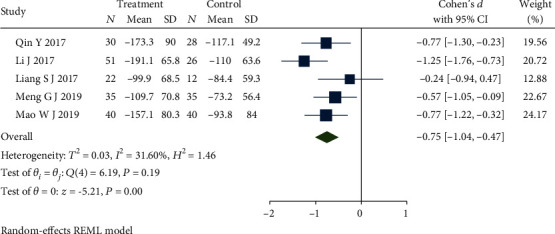
Forest plot of comparison of IBS-SSS between the acupuncture group and dicetel group.

IBS-SSS was reported in 7 studies [35, 36, 39, 43, 44, 46, 49]. Subgroup analysis was completed after it removed one study [49] which used a different oral medication in the control group, but still the obvious heterogeneity among the left studies (*Q*(5) = 107.80, *P* ≤ 0.01, *I*^2^ = 99.60%). Sensitivity analysis and meta-analysis were conducted by removing the study [44] lead to this heterogeneity, which applied a different form of acupuncture that caused the result. The updated forest plot of meta-analysis is shown in Figure 13 and demonstrates that there is low heterogeneity among studies (*Q*(4) = 6.19, *P* = 0.19, *I*^2^ = 31.60%) which used dicetel in the control group, and the difference between the studies is statistically significant (SMD = −0.75, 95% CI [-1.04, -0.47], *P* ≤ 0.01). Combined with shear complement analysis, Egger test results showed that there were no biased publications (*β*_1_ = 3.91, SE of *β*_1_ = 3.81, *z* = 1.03, *P* = 0.30).

### 4.9. Adverse Effect

**Figure 14:**
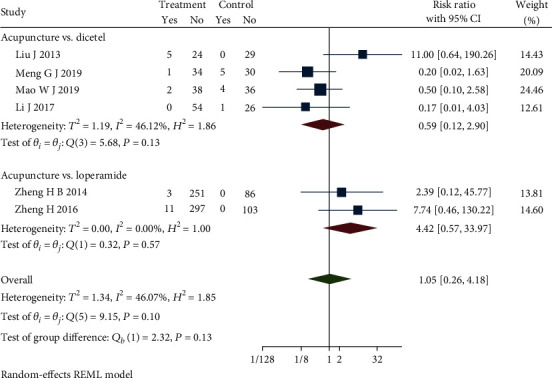
Forest plot of comparison of the adverse effect.

Adverse events were reported in 7 studies [22, 28, 32, 34, 36, 43, 46]. Subgroup analysis completed with these studies, but one subgroup showed an obvious heterogeneity (*Q*(4) = 9.79, *P* = 0.04, *I*^2^ = 61.60%). One study applied a different form of acupuncture that caused the obvious heterogeneity. Sensitivity analysis and then a meta-analysis were carried out after removing the study [22].  Figure 14 presents the updated forest plot and demonstrates that there is no heterogeneity: acupuncture group versus dicetel group (*Q*(3) = 5.68, *P* = 0.13, *I*^2^ = 46.12%) and acupuncture group versus loperamide group (*Q*(1) = 0.32, *P* = 0.57, *I*^2^ = 0.00%). There were more adverse events in the acupuncture group than in the control group. The comparing acupuncture group with dicetel group is no statistically significant (RR = 0.59, 95% CI [0.12, 2.90], *P* > 0.05), but comparing the acupuncture group with the loperamide group is no statistically significant (RR = 4.42, 95% CI [0.57, 33.97], *P* > 0.05). Combined with shear complement analysis, Egger test results showed no biased publications (*β*_1_ = 2.40, SE of *β*_1_ = 2.36, *z* = 1.02, *P* = 0.31).

## 5. Discussion

In this systematic review and meta-analysis, the effectiveness and safety of 31 acupuncture concerned studies for patients with IBS-D or FD were evaluated. We found that acupuncture can significantly reduce the number of stools per week in IBS-D or FD patients, improve patients' overall symptoms, improve the total effective rate, decrease the recurrence rate, and reduce the pain level of patients. Based on the results, we believe that acupuncture can improve the quality of life of patients with IBS-D or FD. Although the number of adverse events in the acupuncture group was similar to that in the control group, the majority of adverse events in the acupuncture group were subcutaneous hemorrhage. With such slight adverse events, we have observed that acceptance among patients has not been reduced. Moreover, the withdrawal rate of patients in the acupuncture group was still slightly lower than that in the control group. Previous studies ignored the importance of the FD which should be related to chronic diarrhea and lack of standard, high-quality clinical trials. This study combined the IBS-D with the FD as the object of research included one standard, high-quality clinical trial [50] which improved the quality of evidence-based medicine. Besides, the patient drop-off rate was reported in our results which showed the comparison of patient receptivity. Unlike previous methods, our study made an advanced analysis through applied the Stata 16.0 software, and some results were evaluated by GRADE that exhibited a more compelling piece of evidence.

The quality of life of IBS-D or FD patients is generally not high that has been demonstrated [51]. Also, the consistency of stool in patients with IBS-D or FD is between type 5 and type 7 on the Bristol stool scale [52]. Among them, abdominal pain is the main diagnostic standard of IBS-D, while FD is mainly diagnosed by excluding the possibility of other diseases [53]. The prevalence of FD and IBS-D in China is 1.72% and 1.54%, respectively [54]. Despite conventional drugs that can temporarily alleviate symptoms, many patients still suffer from the IBS-D or FD, and the recurrence rate was as high as 40% after 3 months. It has been reported that approximately 60.1% of the drug treatment patients stop taking drugs on their own due to the lack of obvious symptom improvement [55, 56]. At present, the etiology and pathogenesis of IBS-D or FD are not clear, but there is growing evidence that pathogenic factors may be related to inflammation, central nervous system disorders, and brain-gut interaction [57]. Serum vasoactive intestinal peptide (VIP) is a neurotransmitter that inhibits gastrointestinal motility and promotes the secretion of intestinal water and electrolytes [58]. 5-hydroxytryptamine (5-HT) as a neurotransmitter also widely exists in the central nervous system and gastrointestinal tract and can regulate gastrointestinal function [59, 60]. Acupuncture, as an alternative therapy for a variety of diseases [61–63], may have achieved the effect of treating IBS-D and FD by regulating nerve-related functions [64]. From the studies included in this review, we also found that acupuncture could improve clinical reports of VIP and 5-HT levels [31].

According to the risk of bias summary and graph, the overall quality of our study is still low. Many studies were regarded as unclear risk of bias in terms of selection bias, performance bias, detection bias, and other bias. Incomplete outcome data in some studies led to a high bias, and we tried to contact authors but got no available datum. The inconsistent diagnostic standards of some studies may lead to the nonstandard diagnosis of FD and IBS-D. Only six studies [22, 32, 34, 48, 50] describe randomized methods and use blinding methods. The remaining studies do not specifically describe randomized or blind treatment methods, which could cause selection bias under the subjective choice of subjects or researchers. And most studies lacked the group of sham acupuncture, and only one study selected the acupuncture plus dicetel compared with sham acupuncture plus dicetel. So, the results of this study were merely a comparison between acupuncture and western medicine, and studies of sham acupuncture groups are still needed. Sensitivity analysis revealed the form of acupuncture, the method of scale scoring, the difference of acupuncture points, and the difference of oral medication in the control group that could be the sources of heterogeneity. In this study, electroacupuncture, warm acupuncture, and eye acupuncture were regarded as the same intervention, even the difference of acupuncture points was hard to keep consistent. Besides, in the clinic, different forms of acupuncture may have different stimulation and patient receptivity. So, potential biases could affect the accuracy of some results. Although our results avoided the high heterogeneity through removed some studies, the reduction in the number of patients affected the quality of the results.

The clinical effect of acupuncture on IBS-D or FD cannot be ignored. It has great safety, can avoid adverse reactions caused by western medicine, and has the advantages of simple operation and low cost [65]. This study objectively explored the effectiveness and safety of acupuncture in the treatment of IBS-D or FD and provided preliminary and reliable evidence-based medicine for clinical practice and decision-making.

## 6. Conclusion

Our systematic review and meta-analysis can prove the effectiveness of acupuncture in the treatment of IBS-D or FD, but it still needs to be verified by a clinical standard large sample test.

## Figures and Tables

**Table 1:** Basic information of the included studies.

Study ID	Sample size (T/C)	Mean age (years)	Diagnostic standards	Intervention	Comparison	Duration (weeks)	Outcome	Adverse effects
Qian et al. 2011 [20]	120 (60/60)	T: 42.5 ± 7.3 C: 43.5 ± 7.6	Roman III	Acupuncture plus dicetel	Sham acupuncture plus dicetel	4	(2)(4)(6)	NR
Sun et al. 2011 [21]	63 (31/32)	T: 38.8 ± 11.8 C: 38.6 ± 11.5	Roman III	Acupuncture	Dicetel	4	(2)(4) (5)(6)	None
Wang et al. 2011 [22]	120 (60/60)	T: 37.2 ± 10.2 C: 40.1 ± 11.7	Roman III	Eye acupuncture	Dicetel	4	(2)(4) (7)(9)	T: 6 C: 0
Chen et al. 2012 [23]	64 (34/30)	T: 41.9 ± 10.0 C: 40.5 ± 8.8	Roman III	Electroacupuncture	Bacillus licheniformis plus deanxit	4	(2)(4)(7)	NR
Li et al. 2012 [24]	64 (32/32)	T: 55.5 ± 5.4 C: 55.3 ± 5.5	Roman III	Acupuncture	Dicetel	4	(2)(4)	NR
Pei et al. 2012 [25]	65 (33/32)	T: 39.1 ± 11.8 C: 37.9 ± 11.5	Roman III	Acupuncture	Dicetel	4	(2)(4)	NR
Wu et al. 2013 [26]	48 (24/24)	T: 41.0 ± 13.0 C: 39.0 ± 13.0	Roman III	Acupuncture	Dicetel	4	(2)(4)(6)	NR
Li et al. 2012 [27]	70 (35/35)	T: 39.1 ± 11.8 C: 37.9 ± 11.5	Roman II	Acupuncture	Dicetel	4	(2)(4) (6)(7)	NR
Liu 2013 [28]	60 (30/30)	T: 37.0 ± 10.1 C: 39.7 ± 10.6	Roman III	Acupuncture	Dicetel	4	(2)(4)(9)	T: 5 C: 0
Zhan et al. 2013 [29]	66 (33/33)	T: 42.5 ± 13.6 C: 37.3 ± 12.7	Roman III	Acupuncture	Dicetel	4	(2)(4) (5)(6)	NR
Wu et al. 2014 [30]	73 (36/37)	T: 39.6 ± 12.8 C: 36.5 ± 14.2	Roman III	Warm acupuncture	Bacillus licheniformis plus deanxit	4	(2)(4)	NR
Li et al. 2014 [31]	60 (30/30)	T: 31.5 C: 33.8	Roman III	Acupuncture	Dicetel	4	(2)	NR
Zheng et al. 2014 [32]	348 (261/87)	T: 41.2 ± 17.1 C: 42.3 ± 18.4	Roman III	Acupuncture	Loperamide	4	(1)(2) (3)(9)	T: 3 C: 0
Li et al. 2015 [33]	40 (24/16)	T: 37.5 ± 16.4 C: 36.9 ± 14.7	Roman III	Electroacupuncture	Loperamide	4	(1)(2) (3)(4)	NR
Zheng et al. 2016 [34]	448 (336/112)	T: 40.5 ± 16.9 C: 40.6 ± 16.7	Roman III	Electroacupuncture	Loperamide	4	(1)(2) (3)(9)	T: 11 C: 0
Qin et al. 2017 [35]	61 (31/30)	T: 41 ± 11 C: 39 ± 12	Roman III	Acupuncture	Dicetel	4	(2)(4)(8)	None
Li et al. 2017 [36]	81 (54/27)	T: 46 ± 13 C: 48 ± 13	Roman III	Acupuncture	Dicetel	6	(2)(4) (8)(9)	T: 0 C: 1
Nie 2017 [37]	100 (50/50)	T: 35.2 ± 6.2 C: 34.2 ± 9.9	Roman III	Acupuncture	Dicetel	6	(2)(4)	NR
Huang 2017 [38]	56 (38/18)	T: 36.3 ± 7.4 C: 38.8 ± 9.9	Roman III	Acupuncture	Dicetel	6	(2)(4)(7)	None
Liang 2017 [39]	34 (22/12)	T: 46.5 ± 11.4 C: 50.8 ± 14.2	Roman III	Acupuncture	Dicetel	6	(2)(4)(8)	NR
Zhong et al. 2018 [40]	60 (30/30)	T: 31.6 ± 12.3 C: 30.2 ± 14.0	Roman III	Electroacupuncture	Loperamide	9	(1)(2)(3)	NR
Yang et al. 2018 [41]	180 (120/60)	T: 40.0 ± 15.4 C: 40.0 ± 15.0	Roman III	Acupuncture	Trimebutine maleate	4	(2)(4)	NR
Zou et al. 2019 [42]	72 (36/36)	T: 42.2 ± 11.2 C: 43.7 ± 12.5	Roman III	Warm acupuncture	Eosinophil-lactobacillus compound tablet	3	(2)(4)(6)	NR
Meng 2019 [43]	70 (35/35)	T: 39.3 ± 11.5 C: 38.4 ± 13.5	Roman IV	Acupuncture	Dicetel	4	(2)(4) (8)(9)	T: 1 C: 5
Zhang 2019 [44]	65 (33/32)	T: 39.5 ± 2.1 C: 39.9 ± 2.1	Roman III	Warm acupuncture	Dicetel	4	(2)(4)(8)	NR
Lu 2019 [45]	76 (38/38)	T: 51.0 ± 9.5 C: 48.0 ± 10.5	Roman III	Acupuncture	Dicetel	4	(2)(4)(6)	NR
Mao 2019 [46]	80 (40/40)	T: 46.4 ± 11.5 C: 47.5 ± 12.4	Roman III	Acupuncture	Dicetel	6	(2)(4)(5) (8)(9)	T: 2 C: 4
Lin 2019 [47]	68 (34/34)	T: 39.9 ± 12.2 C: 40.1 ± 11.2	Compliant with Roman III	Acupuncture plus Dicetel	Dicetel	4	(2)(4)(5)	None
Li 2019 [48]	60 (30/30)	T: 45.0 ± 10.5 C: 45.0 ± 10.0	Roman IV	Warm acupuncture	Dicetel	8	(2)(4)	NR
Liu 2020 [49]	70 (35/35)	T: 42.5 ± 17.5 C: 41.5 ± 8.8	Roman III	Acupuncture	Trimebutine maleate	8	(2)(4)(8)	NR
Li et al. 2020 [50]	392 (261/131)	T: 45.9 ± 13.0 C: 47.0 ± 12.7	Roman III	Acupuncture	Dicetel	6	(2)	NR

**Table 2:** GRADE summary of comparing the acupuncture group with different nonacupuncture groups.

Outcomes	Anticipated absolute effects^∗^ (95% CI)		Relative effect (95% CI)	№ of participants (studies)	Certainty of the evidence (GRADE)
	Assumed risk: nonacupuncture	Corresponding risk: acupuncture			
Weekly defecation	The mean weekly defecation in the control groups was -5.2	The mean weekly defecation in the intervention groups was 0.29 lower (0.49 lower to 0.08 lower)	—	471 (2 RCTs)	⨁⨁⨁◯ MODERATE
Bristol stool form	The mean Bristol stool form in the control groups was -4.16	The mean Bristol stool form in the intervention groups was 0.28 lower (0.68 lower to 0.12 higher)	—	100 (2 RCTs)	⨁⨁◯◯ LOW
Total symptom score	The mean total symptom score in the control groups was -4.1	The mean total symptom score in the intervention groups was 1.17 lower (1.42 lower to 0.93 lower)	—	303 (5 RCTs)	⨁⨁⨁◯ MODERATE
IBS-QOL	The mean IBS-QOL in the control groups was 71.15	The mean IBS-QOL in the intervention groups was 2.37 higher (1.94 higher to 2.80 higher)	—	143 (2 RCTs)	⨁⨁◯◯ LOW
IBS-SSS	The mean IBS-SSS in the control groups was -95.7	The mean IBS-SSS in the intervention groups was 0.75 lower (1.04 lower to 0.47 lower)	—	319 (5 RCTs)	⨁⨁⨁◯ MODERATE

## Supplementary Materials

Supplementary 1 S1: PRISMA Checklist.

Supplementary 2 S2: search strategy of the PubMed database.
