# Supplementary material for: Acupuncture for Adults with Diarrhea-Predominant Irritable Bowel Syndrome or Functional Diarrhea: A Systematic Review and Meta-Analysis
Source: Neural Plast. 2020 Nov 22;2020:8892184. doi: 10.1155/2020/8892184 (PMC7705439; doi:10.1155/2020/8892184)
Supplement: Supplementary 2 — S2: search strategy of the PubMed database. [file 8892184.f2.pdf]

PubMed

#1 "Acupuncture"[Mesh]
#2 "Acupuncture Therapy"[Mesh]
#3 "Acupuncture Analgesia"[Mesh]
#4 "Acupuncture, Ear"[Mesh]
#5 "Electroacupuncture"[Mesh]
#6 "Meridians"[Mesh]
#7 "Acupuncture Points"[Mesh]
#8 "Moxibustion"[Mesh]
#9 acupuncture[Title/Abstract]
#10 need*[Title/Abstract]
#11 stimulat*[Title/Abstract]
#12 eletroacupuncture[Title/Abstract]
#13 point*[Title/Abstract]
#14 prick*[Title/Abstract]
#15 meridians[Title/Abstract]
#16 micmeridians[Title/Abstract]
#17 collaterals[Title/Abstract]
#18 channels[Title/Abstract]
#19 jingluo[Title/Abstract]
#20 ching lo[Title/Abstract]
#21 jing luo[Title/Abstract]
#22 taiyi moxa-cigar[Title/Abstract]
#23 auriculotherapy
#24 OR/1-23
#25 "Diarrhea"[Mesh]
#26 diarrhoea[Title/Abstract]
#27 diarrhea[Title/Abstract]
#28 OR/25-27
#29 "Clinical Trials, Phase II as Topic"[Mesh] OR "Clinical Trials, Phase III as Topic"[Mesh] OR "Clinical Trials, Phase IV as Topic"[Mesh] OR "Controlled Clinical Trials as Topic"[Mesh] OR "Randomized Controlled Trials as Topic"[Mesh] OR "Intention to Treat Analysis"[Mesh]
#30 "Pragmatic Clinical Trials as Topic"[Mesh] OR "Clinical Trials, Phase II"[Publication Type] OR "Clinical Trials, Phase III"[Publication Type] OR "Clinical Trials, Phase IV"[Publication Type] OR "Controlled Clinical Trials"[Publication Type] OR "Randomized Controlled Trials"[Publication Type] OR "Pragmatic Clinical Trials as Topic"[Publication Type]
#31 "Single-Blind Method"[Mesh] OR "Double-Blind Method"[Mesh]
#32 random*[Title/Abstract] OR blind*[Title/Abstract] OR singleblind*[Title/Abstract] OR doubleblind*[Title/Abstract] OR trebleblind*[Title/Abstract] OR tripleblind*[Title/Abstract]
#33 OR/29-32
#34 AND/24,28,33
